# What Next

**Published:** 1889-08

**Authors:** 


WHAT NEXT.—From the reading of a circular lately addressed to the medical profession, and doubtless freely circulated amongst the big lay portion of this community, one would infer that some medical men and women of this city are badly ruptured. We TRUSS'T, however, that such is not the case, and that the amazing *exclamation* of the *duck* shall not be heard by the board of censors.

